# A Plea for Monitoring Serum Selenium Levels in Breast Cancer Patients: Selenium Deficiency Is Rare during the First Year of Therapy, and Selenium Supplementation Is Associated with Elevated Risk of Overdosing

**DOI:** 10.3390/nu16132134

**Published:** 2024-07-04

**Authors:** Laura Alicia Altmayer, Marina Lang, Julia Theresa Schleicher, Caroline Stuhlert, Carolin Wörmann, Laura-Sophie Scherer, Ida Clara Thul, Lisanne Sophie Spenner, Jana Alisa Simon, Alina Wind, Mert Tokcan, Elisabeth Kaiser, Regine Weber, Sybelle Goedicke-Fritz, Gudrun Wagenpfeil, Michael Zemlin, Erich-Franz Solomayer, Jörg Reichrath, Carolin Müller, Cosima Zemlin

**Affiliations:** 1 Department of Gynecology, Obstetrics & Reproductive Medicine, Saarland University, Campus Homburg, 66421 Homburg, Germany; lang.marina@gmx.de (M.L.); juliaschleicher@gmx.de (J.T.S.); caroline.stuhlert@gmail.com (C.S.); carowoer@aol.com (C.W.); laura-sophie.scherer@eurice.eu (L.-S.S.); ida.maier296@googlemail.com (I.C.T.); nelosa@spenner-me.de (L.S.S.); s8jssimo@teams.uni-saarland.de (J.A.S.); alinawind07@gmail.com (A.W.); erich.solomayer@uks.eu (E.-F.S.); carolin.mueller@uks.eu (C.M.); cosima.zemlin@uks.eu (C.Z.); 2 Department of Internal Medicine III-Cardiology, Angiology and Intensive Care Medicine, Saarland University, Campus Homburg, 66421 Homburg, Germany; mert.tokcan@uks.eu; 3 Department of General Pediatrics and Neonatology, Saarland University, Campus Homburg, 66421 Homburg, Germany; elisabeth.kaiser@uks.eu (E.K.); regine.weber@uks.eu (R.W.); sybelle.goedicke-fritz@uks.eu (S.G.-F.); michael.zemlin@uks.eu (M.Z.); 4 Institute for Medical Biometry, Epidemiology and Medical Informatics (IMBEI), Saarland University Campus Homburg, 66421 Homburg, Germany; gudrun.wagenpfeil@uni-saarland.de; 5 Department of Dermatology, Venereology and Allergology, Saarland University, Campus Homburg, 66421 Homburg, Germany; joerg.reichrath@uks.eu; 6 Outcomes Research Consortium, Department of Anesthesiology, Cleveland Clinic, Cleveland, OH 44195, USA

**Keywords:** selenium, breast cancer, nutrition, complementary medicine, body composition, antitumoral therapy

## Abstract

(1) Background: The role of selenium in cancer biology remains poorly understood. Our aim was to study the course of selenium serum levels and the use of selenium supplements during breast cancer therapy. (2) Methods: Serum selenium levels, clinical–pathological data, selenium supplementation, and lifestyle factors were monitored quarterly over one year. (3) Results: A total of 110 non-metastatic breast cancer patients were enrolled in the prospective observational “BEGYN-1” study. At baseline, 2.9% of patients were selenium-deficient (<50 ng/mL), 1.9% were overdosed (>120 ng/mL), and 6.4% received substitution. The median selenium level was 81.5 ng/mL and ranged between 78.7 and 84.5 ng/mL within the year. A total of 25.3% of the patients received supplementation, resulting in significantly higher selenium levels (*p* < 0.05). A total of 8.7–28.6% of the patients using supplements were overdosed. Selenium levels strongly correlated with mushroom consumption (*p* = 0.003), but no association was found with therapy or clinical characteristics. (4) Conclusions: Although selenium deficiency is rare, serum selenium levels should be assessed in breast cancer patients. Mushrooms and nuts should be preferred over supplements to correct selenium deficiency. Ruling out selenium deficiency helps prevent the risk of selenosis and avoid unnecessary, costly supplementation in patients who are often financially burdened due to their disease.

## 1. Introduction

Breast cancer is the most common cancer worldwide, with an estimated 2.3 million cases in 2020, and is the leading cause of cancer-related death in women [1,2]. Research is underway to improve prevention, diagnosis, and treatment strategies. However, patients themselves also wish to contribute to the success of treatment. Despite questionable benefits, the use of dietary supplements by oncologic patients has increased in recent years because many patients believe that taking micronutrients or vitamins could improve their health [3]. The prevalence of supplement use among oncologic patients varies considerably, with estimates ranging from 30 to 90% in different studies. These patients often take supplements or presumed immunoprotective micronutrients without their physician’s knowledge [3,4]. For instance, the SWOG 0221 study assessed the use of dietary supplements in 1467 breast cancer patients before diagnosis and during treatment [5]. A total of 48.1% of the study population were taking multivitamins prior to being diagnosed with breast cancer [5]. However, the efficacy or influence of multivitamins on oncological disease and treatment is controversial [3,6,7]. Furthermore, many patients take supplements without knowing whether they are deficient, resulting in the risk of overdosing. One micronutrient frequently used in this context is selenium. Selenium is a ubiquitous, nutritionally essential trace element in the human body. In the form of the amino acid selenocysteine, selenium is a component of numerous enzymes and proteins [8]. It plays an important role in protecting cells from oxidative stress and regulating cell growth and thyroid hormone metabolism [8,9,10,11,12]. Compared to other organs, the thyroid gland has the highest selenium content [12,13]. It is also thought to have an anti-inflammatory effect through its influence on eicosanoid metabolism [14].

Humans obtain selenium almost exclusively from food, and adequate selenium intake is possible through a balanced diet [8,15]. Protein-rich foods such as meat, fish, mushrooms, eggs, nuts, and cereals are particularly rich in selenium [8,16]. Milk, cheese, vegetables, and fruit also contain small amounts of selenium [8,16]. The amount of selenium in different foods can vary widely [8]. Brazil nuts are one of the richest sources of selenium, containing between 1 and 20 mg of selenium per kilogram [12,15]. Selenium is non-specifically incorporated into body proteins in the form of selenomethionine, particularly in organs with a high rate of protein synthesis, such as skeletal muscle, liver, or kidneys, where it is stored. By converting selenomethionine to selenocysteine, selenium can be mobilized for metabolic processes in the liver or kidneys [17].

The German Nutrition Society recommends a daily selenium intake of 60 µg for women and 70 µg for men [18,19]. A daily selenium intake of 300–450 µg for adults has been suggested by various institutions (Scientific Committee on Food, Expert Group on Vitamins and Minerals, Food and Nutrition Board of the U.S. National Academy of Sciences, Institute of Medicine) as a safe upper limit, at which no adverse effects should occur [8,12,15,20]. 

Selenium deficiency may occur if less than 20 µg of selenium per day is consumed over a prolonged period [8]. Toxic side effects can occur with a daily intake of 900 to 1200 µg [12,20,21]. Both persistent selenium deficiency and selenium overdose may be associated with non-specific health complaints [12,20,22]. These include gastrointestinal and muscular symptoms, fatigue, reduced performance, immune system, and thyroid dysfunction [8,12,22]. Acute selenosis caused by the ingestion of several grams of selenium can lead to heart failure, ventricular fibrillation, and death [15]. One common cause of selenosis is accidental or deliberate overuse of dietary supplements [23].

It is crucial to emphasize that chronic exposure to high selenium doses, often due to misformulated dietary supplements, can also result in selenium toxicity, selenosis symptoms, and adverse health effects [24,25].

It has been shown that chronic selenotoxicity can occur even at levels of selenium intake previously considered harmless, thereby increasing the risk of chronic disease [24]. This calls into question the widespread use of selenium supplements [24].

The causality between selenium deficiency and various diseases, as well as the anti-carcinogenic effect of selenium, have been discussed since the 1960s [26,27]. As a component of many enzymes, Selenium is considered to be of great functional importance in the human body. It is often used as a supportive measure in many diseases, including cancer, because it is thought to have a tumor-protective and anti-proliferative effect, contributing to the inhibition of tumor growth and having an inhibitory effect on the development of metastases and recurrence [28,29,30]. 

In Europe, including Germany, healthy people have mean selenium serum levels of about 84 ng/mL (reference range 50–120 ng/mL [8]), whereas some studies observed lower selenium serum concentrations below 70 ng/mL in cancer patients [3]. Lower selenium levels have also been associated with poorer overall survival in breast cancer patients [31]. 

In strong contrast, a Cochrane review found that selenium supplementation had no effect on reducing the overall risk of cancer or the risk of certain types of cancer [32]. There was even evidence that selenium may increase the risk of high-grade prostate cancer, type 2 diabetes, and skin disorders [32]. A higher risk of breast cancer has also been found with increased selenium exposure [32]. The German S3 guideline on complementary medicine in oncology does not recommend selenium supplementation for breast cancer patients [7]. Further research has shown that the influence of selenoproteins changes both during the carcinogenic process and in a tissue-specific manner [33]. During the initiation phase, selenoproteins protect cells from oxidative DNA damage and thus appear to inhibit tumor development, while in existing tumor cells, selenoproteins support their growth [33].

Rodemann et al. described in their in vitro experiments that selenium has a radioprotective effect on healthy body cells but not on tumor cells [34]. Some studies observed a decrease in serum selenium levels after radiotherapy in breast cancer [35]. Selenium supplementation for chemoprevention reduced the risk of chromosome breaks in patients with BRCA1 mutations to the level of unaffected patients, thus preventing the risk of cancer [36].

It is also hypothesized that high selenium intake is associated with favorable body composition. Dietary intake of selenium is approximately 30% lower in obese patients than in normal-weight individuals. A dose-dependent inverse relationship has been found between selenium intake and BMI or total body fat [37,38].

Existing data on the role of selenium in tumor patients, particularly those with breast cancer, are contradictory [3,8,32]. Furthermore, the general occurrence of selenium deficiency and overdosage is not exactly known, as selenium testing is not part of the clinical routine [6,7]. In the present study, we aimed to analyze serum selenium levels during oncological therapies (e.g., chemotherapy, radiotherapy, endocrine therapy, HER2-targeted therapy) and how selenium supplementation, as well as lifestyle factors and nutrition, contributed to these changes during the first year after diagnosis of non-metastatic breast cancer. 

## 2. Materials and Methods

### 2.1. Data Collection

From September 2019 to January 2022, the BEGYN-1 study prospectively observed 110 non-metastatic breast cancer patients at the Saarland University Medical Center. The study received approval from the Ethical Committee of the Medical Association of Saarland (study # 229/18, date of approval 6 November 2019). The study included female participants aged 18 years or older who had invasive non-metastatic breast cancer, possessed adequate German language skills to complete questionnaires and activity diaries, and had sufficient technical skills or support to use a smartphone and fitness tracker. In addition, all patients had to provide written informed consent. Patients were excluded if they had received oncological treatment prior to baseline, had non-invasive disease (e.g., carcinoma in situ), had a current history of other neoplasia, had a life expectancy of less than 12 months, were unable to perform treadmill spiroergometry, or were pregnant or breastfeeding.

Serum selenium levels were measured using atomic absorption spectrometry at the Medical Care Centre Laboratory Volkmann in Karlsruhe, Germany. Measurements were conducted at the baseline visit, prior to the start of any pharmacological, radiological, or surgical therapy, and subsequently every three months during the first year following the initial diagnosis. At each visit, patients were asked about supplementation of vitamins or trace elements (including selenium). The supplementation and dosages of vitamins were then documented (at 0, 3, 6, 9, and 12 months). Patients received selenium substitution according to their test results. 

Other laboratory values (including vitamin D serum levels, blood lipids, thyroid hormones, T cell subpopulations, and plasma cytokines) were collected every three months and analyzed in a routine clinical laboratory. At the quarterly visits, bioelectrical impedance analysis was performed using the TANITA BC-601 scale TM (Tanita Europe BV, Stuttgart, Germany). Moreover, patients underwent sprioergometry on a treadmill every three months, and information on physical activity was obtained using a fitness tracker and an activity diary. The results have previously been published [39].

Furthermore, information on clinicopathological data was retrieved from the hospital’s digital documentation system (SAP, Walldorf, Germany). Nutritional data were obtained through a questionnaire administered during the baseline visit. The data collection process was previously described in detail in the BEGYN study protocol [40,41].

### 2.2. Statistics

Statistical analyses were performed using IBM^®^ SPSS^®^ (Statistical Package for the Social Sciences) Statistics, version 27.0 (International Business Machines Corporation, Armonk, NY, USA). Sample size calculation was previously presented in our published study protocol [40,41]: A sample size of 110 produces a two-sided 95% confidence interval with a distance from the mean to the limits that is equal to 2.835 when the estimated standard deviation is 15.0. A sample size of 110 produces a two-sided 95% confidence interval with a width equal to 0.187 when the sample proportion is 0.50.

Qualitative parameters are presented as absolute and percentage frequencies. All variables were tested for normal distribution using Kolmogorov–Smirnov or Shapiro–Wilk test. Some parameters were not normally distributed. Therefore, in the following, quantitative parameters are uniformly expressed as median with minimum and maximum, and non-parametric tests, i.e., Mann–Whitney U test or Kruskal–Wallis test, were uniformly used for comparisons between two or more independent groups. Correlations were calculated using Spearman’s correlation. To investigate the influence of substitution in more detail, a univariate analysis with generalized estimating equations (GEE) was performed, considering repeated measurements.

## 3. Results

At baseline, BEGYN-1 enrolled 110 patients who were assessed every 3 months for one year (0 months = baseline, 3, 6, 9, and 12 months) after their breast cancer diagnosis. Nineteen patients withdrew from the study over the course of the year. Previously published data on tumor stage are available [40,41]. The median age of the patients was 55 years (minimum 26, maximum 81). There was no significant correlation between selenium level and age (r = −0.15; *p* = 0.13). Table 1 displays data on clinical characteristics, including tumor entity, UICC/AJCC stage, grading, tumor biology, progesterone receptor and estrogen receptor status, mutations, and menopause, and their association with serum selenium levels. 

**Figure 1:**
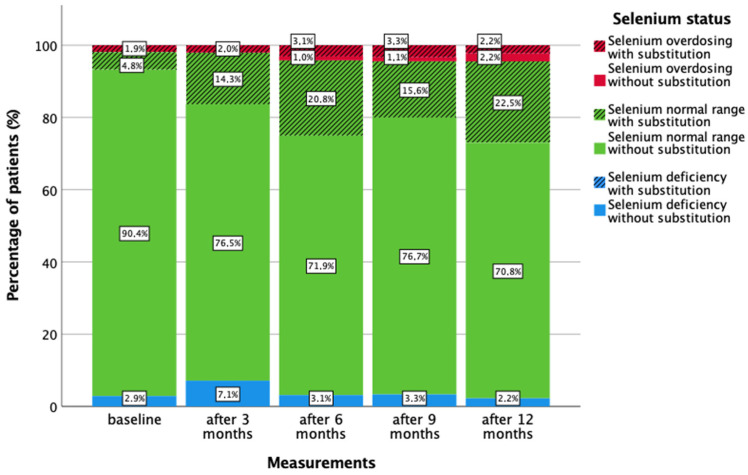
Serum selenium status throughout the year (0, 3, 6, 9, and 12 months), considering the substitution of selenium.

**Figure 2:**
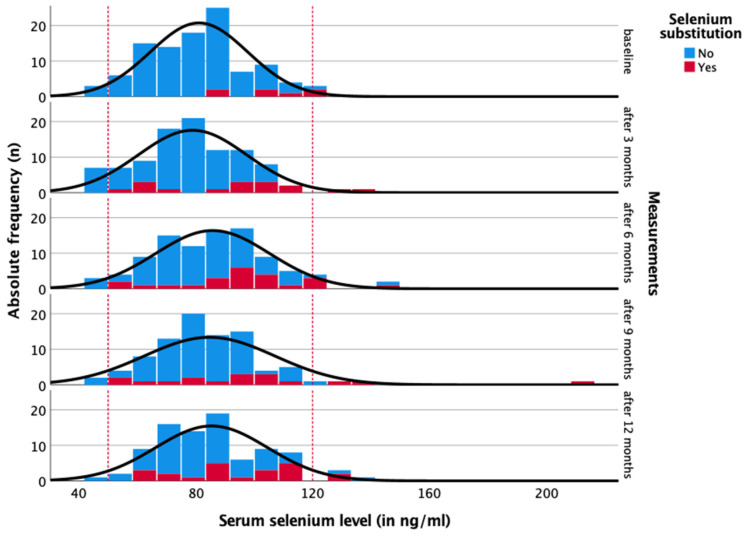
Serum selenium levels throughout the year (0, 3, 6, 9, and 12 months) considering the substitution of selenium. Reference range in dotted lines (50–120 ng/mL).

Prior to treatment, 95.2% of patients had serum selenium levels within the normal range, with a median of 81.5 ng/mL (44.4–123.2 ng/mL; Figure 1 and Figure 2 and Table 2). Only 2.9% of patients suffered from a selenium deficiency of less than 50 ng/mL (minimum: 44.4 ng/mL), and 1.9% had an overdose of up to 123.2 ng/mL at baseline. Selenium substitution was administered to 6.4% of patients. At all five measurement points, more than 90% of patients had serum selenium levels within the recommended normal range of 50–120 ng/mL [8]. Selenium deficiency was most common three months after diagnosis, affecting 7.1% of patients, with a median serum level of 78.7 ng/mL (41.9–139.3 ng/mL). At 12 months, 24.7% of patients were receiving selenium supplementation, 4.4% had an overdose, and only 2.2% had a deficiency. None of the patients experienced a deficiency while on supplementation. Table 3 presents the amount of selenium substitution during the year (0 months = baseline, 3, 6, 9, and 12 months). Patients who received selenium supplementation had significantly higher serum selenium levels than those who did not (*p* < 0.05), with 1.9 to 4.4% of patients per measurement showing a selenium overdose above 120 ng/mL. Substituting 1 µg of selenium per day increased selenium levels by 0.11 ng/mL (*p* < 0.001). This means that a patient who substituted 100 µg per day had an average increase in serum selenium levels of 11 ng/mL over time.

**Figure 3:**
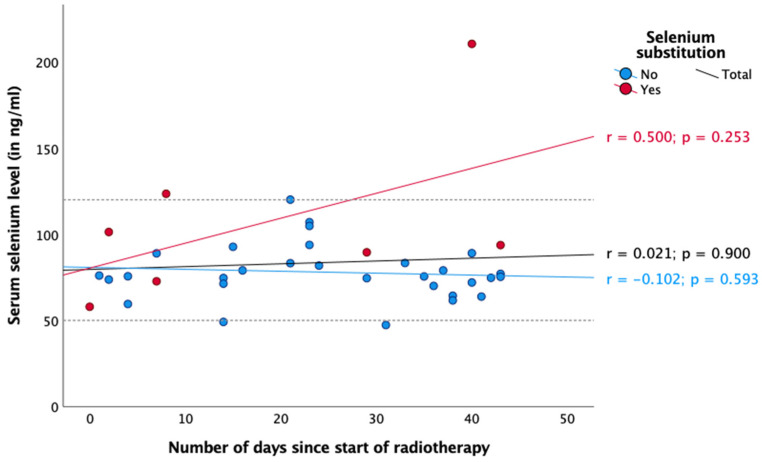
Serum selenium levels in patients receiving radiotherapy. Reference range in dotted lines (50–120 ng/mL). Spearman correlation was performed between serum selenium levels and number of days since the start of radiotherapy with r = correlation coefficient and with a significance level of *p* < 0.05.

**Figure 4:**
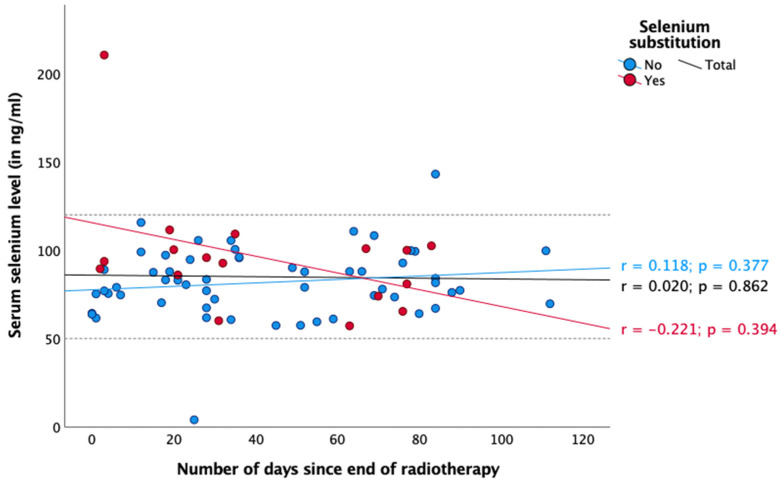
Serum selenium levels after all patients finished radiotherapy. Reference range in dotted lines (50–120 ng/mL). Spearman correlation was performed between serum selenium levels and number of days since the end of radiotherapy with r = correlation coefficient and with a significance level of *p* < 0.05.

**Figure 5:**
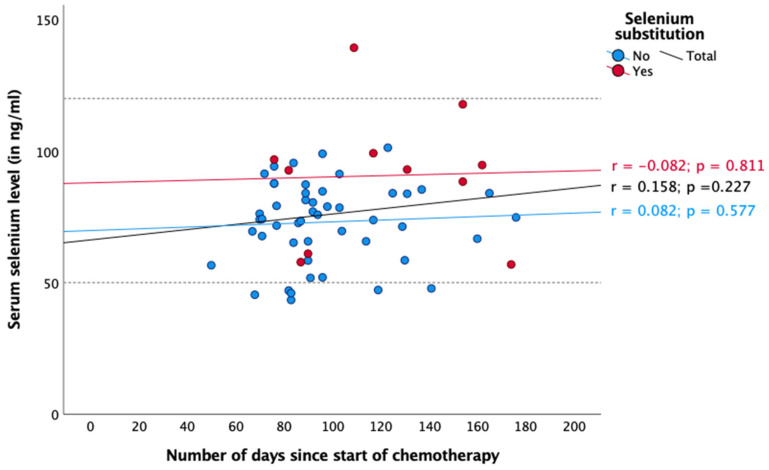
Serum selenium levels in patients receiving chemotherapy. Reference range in dotted lines (50–120 ng/mL). Spearman correlation was performed between serum selenium levels and number of days since the start of chemotherapy with r = correlation coefficient and with a significance level of *p* < 0.05.

**Figure 6:**
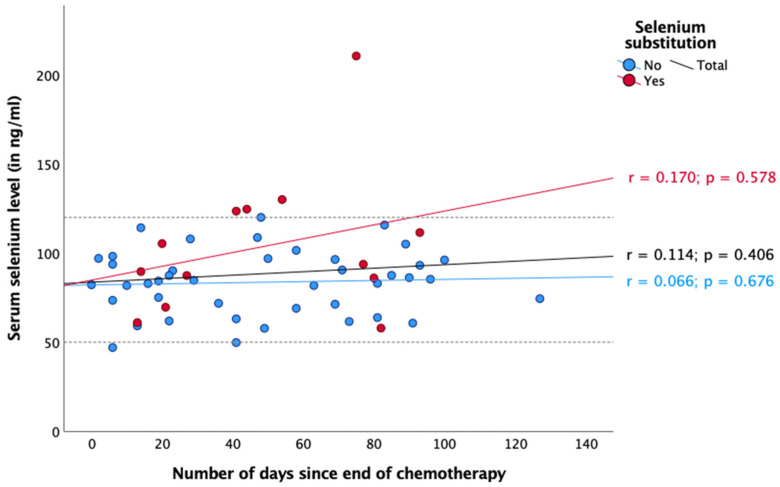
Serum selenium levels after chemotherapy. Reference range in dotted lines (50–120 ng/mL). Spearman correlation was performed between serum selenium levels and number of days since the end of chemotherapy with r = correlation coefficient and with a significance level of *p* < 0.05.

**Figure 7:**
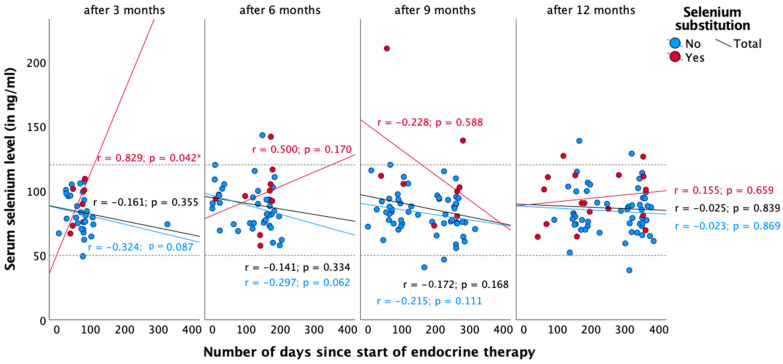
Course of serum selenium levels in patients receiving endocrine therapy. Reference range in dotted lines (50–120 ng/mL). Spearman correlation was performed between serum selenium levels and the number of days since the start of endocrine therapy with r = correlation coefficient and with a significance level of *p* < 0.05. * = *p* < 0.05.

Regarding radiotherapy and chemotherapy, there was no significant association between selenium status and the number of days since the start or end of the respective therapy (Figure 3, Figure 4, Figure 5 and Figure 6). In the case of endocrine therapy, it was observed that selenium levels decreased with the duration of the endocrine therapy in non-substituting patients. However, this observation was not significant (Figure 7).

Additionally, during the baseline visit, patients were questioned about their dietary habits, as shown in Table 4. A significant positive correlation was found between selenium levels and mushroom consumption (r = 0.324, *p* = 0.003). However, no significant correlation was observed between selenium levels and the consumption of fish, eggs, milk products, cheese, or butter.

Table 5 displays the correlation between serum selenium levels and body composition measured by bioelectrical impedance analysis. No significant correlation was found between selenium and body composition, including weight, BMI, body fat percentage, visceral fat, muscle mass, and bone mass.

Additionally, Table 6 shows no significant association between selenium and thyroid hormones (TSH, T3, and T4).

## 4. Discussion

In the present study, the median serum selenium level of patients was 81.5 ng/mL. The majority of patients (95.2%) had selenium levels within the recommended reference range of 50 to 120 ng/mL [8]. In other observational and case–control studies in breast cancer patients, a mean selenium level of 86.2 ng/mL [31] and 81.1 ng/mL [42] was measured. Compared with the healthy European general population (84 ng/mL) [3] and the German general population (70–80 ng/mL) [8], the level does not appear to be substantially different [3]. Thus, our study does not support the hypothesis that the risk for breast cancer would be associated with lower serum selenium levels. 

At baseline, 6.4% of patients were using selenium supplements. During the first year, this percentage increased remarkably to 25.3%. This increase may be attributed to recommendations from physicians, friends, or relatives [43], as well as increased advertising by the pharmaceutical industry regarding the importance of taking selenium after a cancer diagnosis. 

Most patients without substitution were within the normal range during treatment. In accordance with the recommendation of the German S3 guideline on oncology-complementary medicine [7], general substitution during therapy is not recommended for breast cancer patients. Instead, it might be beneficial to assess serum selenium levels at diagnosis of breast cancer as patients with normal selenium levels can be reassured that substitution is unnecessary and should be avoided to prevent overdosing and selenosis. Patients with selenium deficiency may benefit from substitution or a dietary modification to include selenium-rich food such as mushrooms or nuts. Moreover, an overdose of selenium could be identified by a routine control of serum selenium levels at diagnosis of breast cancer. In the present study, some patients who took selenium supplements, and even some who reported not taking selenium supplements, had a selenium overdose. It is therefore reasonable to assume that unnecessary selenium supplementation may lead to selenium overdose and undesirable side effects [8,12,22]. In our opinion, it seems sensible to recommend a determination of baseline selenium level at diagnosis of breast cancer. In contrast, only patients with selenium deficiency or overdose should undergo regular laboratory tests until their levels normalize.

Some patients were observed who had a low selenium baseline value in the deficiency range and whose selenium levels rose into the normal range after supplementation. However, the selenium deficiency reoccurred after supplementation was discontinued. This suggests that selenium levels should be monitored, especially in patients who have previously been diagnosed with selenium deficiency. Lifelong supplementation or a change in diet may be necessary. However, even a sufficient selenium baseline value does not guarantee that selenium levels will not fall into the deficiency range. It seems that tests could help to detect selenium deficiency in specific indications, and a diet rich in selenium, such as mushrooms and nuts (in moderation), could be useful in preventing selenium deficiency.

Three months after diagnosis, the proportion of patients with selenium deficiency increased from 2.9% to 7.1%, and the median selenium level fell from 81.5 to 78.7 ng/mL (*p* = 0.360). However, the number of patients who have substituted had increased (0 months: 6.4%, 3 months: 16.8%). Over the course of the year, the median serum selenium levels increased to 84.3 ng/mL after one year. The changes did not reach statistical significance (*p* = 0.078). Nevertheless, we sought to identify potential explanations for the observed changes in serum selenium levels. With regard to radiotherapy, chemotherapy, and endocrine therapy, no significant association was observed between selenium status and the number of days since the start and end of therapy, respectively (Figure 4, Figure 5, Figure 6 and Figure 7). 

It is noteworthy that the mean selenium concentration exhibited a slight increase during chemotherapy (Figure 5 and Figure 6), whereas during radiotherapy, data were inconsistent (Figure 3 and Figure 4). None of our observations showed any significance. Nevertheless, a potential explanation for this phenomenon could be that patients consumed foods containing selenium (e.g., Brazil nuts) during chemotherapy, either in accordance with their physician’s advice or on their own initiative, which could explain the observed increase in selenium levels.

During endocrine therapy, patients without selenium supplementation had lower selenium levels over the year (Figure 7). There is currently no scientific evidence of a relationship between selenium and endocrine therapy. Patients receiving selenium supplementation showed inconsistent courses, probably due to the very small number of cases and due to outliers. A possible explanation for the inconsistent serum selenium levels could also be that patients consumed different foods containing selenium (e.g., Brazil nuts) and unknown supplements during therapy, which could explain the changes in serum selenium levels. Therefore, examining the selenium level can be useful. 

In a randomized controlled trial, some participants received a total of 13 g of defatted, granulated Brazil nuts per day, equivalent to approximately four Brazil nuts and approximately 227.5 μg of selenium per day [44]. The intake of Brazil nuts resulted in a significant increase in selenium levels from 87.0 ± 16.8 to 180.6 ± 67.1 ng/mL (an increase of 119%) within 12 weeks [44]. From this, it can be concluded that a deficiency can be easily corrected through a simple change in diet by adding, e.g., two Brazil nuts per day [15,45]. On the other hand, patients whose serum selenium levels are within the normal range can be reassured that supplementation is not necessary, thus preventing them from spending money unnecessarily, as they are often already financially burdened by the disease.

According to our results, patients who consumed mushrooms on more days per month had higher selenium levels than those who consumed little or no mushrooms (r = 0.324, *p* = 0.003). Mushrooms are known to be rich in selenium [8]. Eating foods that contain selenium, such as mushrooms and Brazil nuts, can help maintain selenium levels in the normal range or increase the selenium level from the deficiency range to the normal range. This means that supplementation may not be absolutely necessary in cases of selenium deficiency and that a deficiency could be corrected more naturally by dietary changes. Therefore, patients would often save huge amounts of money. It is known that approximately 25 to 33% of gynecological patients suffer from financial problems [46,47]. Cancer can reduce people’s ability to work, leading to reduced working hours, job loss, or early retirement [48]. On average, cancer patients in Germany lose 26% of their income [48]. This can lead to financial strain and financial toxicity, which can have a considerable impact on quality of life and possibly also on the prognosis of the disease [48]. The purchase of dietary supplements is associated with additional costs for patients who are already financially burdened. 

The producers heavily promote their pharmaceutical products. They argue that they can help improve the patients’ wellbeing and sell them as absolutely necessary. Notably, research has shown that the pharmaceutical industry spends twice as much money on advertising as it does on research [49]. Furthermore, the advertising strikes a fertile ground as patients attempt to regain lost control over their health condition. Critical examination of pharmaceutical industry advertising and raising awareness of this issue among patients and physicians should be at the forefront. It is time to enforce regulations that prevent misleading advertising of supplements with unknown effects.

We hypothesize that it is more beneficial to obtain selenium from the diet than from expensive supplements. Further randomized controlled trials comparing a selenium-rich diet with selenium supplements should be conducted.

Overall, we could not find a link between breast cancer and selenium. We cannot confirm the suggestion that the change in serum selenium concentration in women with breast cancer is most likely a consequence rather than a cause of the disease or therapeutic interventions [42]. According to our results, a change in selenium concentration is neither a cause nor a consequence of the disease. It should be emphasized that only mild selenium deficiency was found in the present study. The lowest selenium value was 38.4 ng/mL, and all other values in the selenium deficiency range were between 40 and 49.9 ng/mL. The overall change is rather small, and at all five time points, more than 90% of the patients were within the recommended selenium range. The percentage of selenium deficiency is therefore low and not necessarily due to breast cancer but can also be explained by diet. It can be assumed that there is also a small proportion of patients with selenium deficiency or excess in the general German population. In addition to studies examining selenium intake in the general population, more studies should be conducted to determine selenium status in serum.

One of the limitations of the study is that selenium levels were measured independently of therapy. Additionally, compliance and patients’ self-reported information on supplementation and dietary habits cannot be independently verified, e.g., some patients take mixed preparations, including selenium, without knowing exactly which active ingredients they contain. Despite careful investigation and the development of a relationship of trust, it cannot be ruled out with 100% certainty that patients were taking selenium supplementation without our knowledge. Furthermore, we did not explicitly ask for the patients’ consumption of Brazil nuts or other nuts. In addition to patients’ self-reports, varying selenium levels in different food sources may also have caused inaccuracies. Selenium is naturally present in soil, influencing the selenium content of plant-based foods. In regions with sufficient soil selenium levels, excellent organic sources of selenium include Brazil nuts, green vegetables, and button mushrooms [8,12,15,16]. Moreover, the absorption rate of selenium by plants is crucial and is determined by a number of factors, including plant type, soil pH levels, soil composition, rainfall patterns, microbial activity, and other biogeochemical elements [50]. For this reason, not only diet and reported dietary habits but also food per se plays a crucial role in determining selenium levels in patients. Which foods (from which manufacturer, etc.) the patients consumed and how much selenium the individual foods actually contained were not analyzed as part of the BEGYN-1 study. Therefore, dietary inaccuracies might have influenced our research results. Overall, research on selenium and cancer is complicated by the existence of a large number of organic and inorganic selenium compounds, each with different biological properties, as well as various lifestyle and dietary factors that are difficult to objectify [32]. Furthermore, genetic predispositions or environmental exposures that might have an influence on selenium serum levels were not assessed within the BEGYN-1 study. 

## 5. Conclusions

Although selenium deficiency is rare in breast cancer patients, we recommend monitoring the selenium levels in newly diagnosed breast cancer patients. A deficiency can be easily corrected through nutritional supplements or a change in the diet by adding, e.g., two Brazil nuts per day [15,44,45]. On the other hand, patients with serum selenium levels within the normal range can be reassured that supplementation is unnecessary and should be avoided to prevent overdosing and selenosis. This reduces their financial burden and encourages cancer patients to focus on more important therapies, such as physical activity. Further studies with larger sample sizes are needed to investigate the effects of selenium deficiency in breast cancer patients and during oncological treatment, considering the influence of various forms of supplementation, lifestyle, genetic predisposition, and dietary factors in particular.

## Figures and Tables

**Table 1:** Median serum selenium levels by clinical characteristics.

Clinical Characteristics		n	%	Serum Selenium Level (in ng/mL)	*p*-Value
Tumor entity	NST	91	82.7%	84.0 (44.4–123.2)	0.858
	Invasive lobular	12	10.9%	81.5 (52.0–108.7)	
	Others ^1)^	7	6.4%	83.9 (62.2–110.3)	
UICC-/AJCC stage	I	46	41.8%	84.0 (44.4–123.2)	0.629
	II	56	50.9%	78.3 (52.0–120.8)	
	III	8	7.3%	82.4 (46.1–93.3)	
Grading	G1	10	9.2%	83.8 (62.2–110.3)	0.506
	G2	56	51.4%	80.6 (44.4–116.9)	
	G3	43	39.4%	84.2 (52.0–123.2)	
Tumor biology	Luminal A	48	43.6%	81.2 (44.4–110.3)	0.497
	Luminal B	24	21.8%	79.6 (57.3–123.2)	
	HER2/neu-positive	27	24.5%	78.3 (46.1–116.9)	
	Triple-negative	11	10.0%	85.9 (54.9–102.2)	
ER status	Positive	89	80.9%	81.5 (44.4–123.2)	0.811
	Negative	21	19.1%	81.1 (54.9–102.2)	
PR status	Positive	72	65.5%	80.4 (44.4–123.2)	0.325
	Negative	38	34.5%	84.2 (54.9.1–116.9)	
Mutations	No	30	71.4%	79.6 (44.4–114.6)	0.070 ^2)^
	BRCA1/2	8	19.1%	89.0 (66.2–123.2)	
	CHECK/ATM	4	9.5%	69.6 (64.1–74.6)	
Menopause	No	33	30.0%	81.5 (52.0–120.8)	0.464
	Yes	77	70.0%	81.2 (44.4–123.2)	

n = absolute number of patients. % = percentage of patients. Serum selenium levels are given as median with minimum and maximum. For *p*-value, the Mann–Whitney U test was performed for two independent groups or the Kruskal–Wallis test was performed for more than two independent groups with a significance level of *p* < 0.05. ^1)^ Inflammatory, mucinous, tubular, metaplastic, mixed (NST, tubular). ^2)^ CHECK/ATM was excluded from the calculations due to the small group size (*n* = 4).

**Table 2:** Median serum selenium levels throughout the year (0, 3, 6, 9, and 12 months), frequency of substitution, and overdose with substitution.

Measurements	Serum Selenium Level in ng/ml	Frequency of Substitution	Overdose with Substitution
Baseline	81.5 (44.4–123.2)	7 of 110 (6.4%)	2 of 7 (28.6%)
After 3 months	78.7 (41.9–139.3)	17 of 101 (16.8%)	2 of 17 (11.8%)
After 6 months	84.5 (47.2–143.1)	23 of 96 (24.0%)	3 of 23 (13.0%)
After 9 months	82.4 (40.7–210.5)	17 of 90 (18.9%)	3 of 17 (17.6%)
After 12 months	84.3 (38.4–138.7)	23 of 91 (25.3%)	2 of 23 (8.7%)

Serum selenium levels are given as median with minimum and maximum.

**Table 3:** Dosage of selenium substitution throughout the year (0, 3, 6, 9, and 12 months).

Dosage	0 Months *n* = 7/110 (6.4%)	3 Months *n* = 17/101 (16.8%)	6 Months *n* = 23/96 (24.0%)	9 Months *n* = 17/90 (18.9%)	12 Months *n* = 23/91 (25.3%)
100 µg/day	0 (0.0%)	3 (17.6%)	3 (13.0%)	2 (11.8%)	3 (13.0%)
200 µg/day	1 (14.3%)	2 (11.8%)	4 (17.4%)	3 (17.6%)	5 (21.7%)
300 µg/day	1 (14.3%)	1 (5.9%)	6 (26.1%)	4 (23.5%)	7 (30.4%)
50 µg/day	0 (0.0%)	2 (11.8%)	2 (8.7%)	0 (0.0%)	1 (4.3%)
30 µg/day	0 (0.0%)	0 (0.0%)	0 (0.0%)	0 (0.0%)	1 (4.3%)
Variable, 100–300 µg/day	0 (0.0%)	1 (5.9%)	1 (4.3%)	1 (5.9%)	1 (4.3%)
35 µg/day LaVita juice (1 tbsp)	0 (0.0%)	2 (11.8%)	2 (8.7%)	3 (17.6%)	4 (17.4%)
113 µg Sinekrin (2 tablets)	1 (14.3%)	1 (5.9%)	1 (4.3%)	1 (5.9%)	2 (8.7%)
No information on exact dosage	4 (57.1%)	5 (29.4%)	6 (26.1%)	3 (17.6%)	1 (4.3%)

**Table 4:** Dietary habits and selenium content in various food products.

Various Food Products	Dietary Intake (in Days per Month)	Selenium Content (in μg/kg)	Spearman Correlation
Herring/trout/salmon	3.0 (0–12)	250–430	r = 0.044 *p* = 0.694
Mackerel/tuna	1.0 (0–8)	390–820	r = 0.132 *p* = 0.239
Eggs/margarine	12.0 (0–28)	Eggs: 100–200 Margarine: no data available	r = 0.104 *p* = 0.355
Cream/gouda/butter	20.0 (0–28)	No data available	r = 0.003 *p* = 0.978
Whole milk/quark/yogurt	20.0 (0–28)	5–20	r = 0.156 *p* = 0.166
Chanterelles/champignons/ porcini mushrooms	3.0 (0–28)	10–1800	r = 0.324 *p* = 0.003
Beef/veal liver	0 (0–5)	20–200	r = 0.198 *p* = 0.077

Dietary intake is given as median with minimum and maximum. Spearman correlation was performed between serum selenium levels and dietary intake of different foods with r = correlation coefficient and with a significance level of *p* < 0.05. Patients with selenium substitution were excluded.

**Table 5:** Spearman correlation between serum selenium level and body composition at different points in time within one year after breast cancer diagnosis.

Body Composition	Selenium Level at Baseline	Selenium Level after 3 Months	Selenium Level after 6 Months	Selenium Level after 9 months	Selenium Level after 12 Months
Weight	r = −0.06 *p* = 0.52	r = 0.05 *p* = 0.63	r = 0.07 *p* = 0.49	r = 0.10 *p* = 0.35	r = 0.06 *p* = 0.61
Body Mass Index	r = −0.12 *p* = 0.24	r = −0.01 *p* = 0.89	r = 0.11 *p* = 0.30	r = 0.12 *p* = 0.26	r = 0.07 *p* = 0.50
Muscle mass	r = 0.04 *p* = 0.677	r = 0.04 *p* = 0.69	r = −0.01 *p* = 0.94	r = −0.08 *p* = 0.44	r = 0.06 *p* = 0.60
Body fat percentage	r = −0.14 *p* = 0.16	r = 0.07 *p* = 0.52	r = 0.15 *p* = 0.16	r = 0.20 *p* = 0.06	r = 0.07 *p* = 0.54
Visceral fat	r = −0.17 *p* = 0.08	r = −0.02 *p* = 0.84	r = 0.14 *p* = 0.18	r = 0.09 *p* = 0.40	r = 0.09 *p* = 0.41
Bone mass	r = 0.05 *p* = 0.63	r = 0.05 *p* = 0.61	r = −0.02 *p* = 0.85	r = −0.09 *p* = 0.40	r = 0.02 *p* = 0.84

Spearman correlation was performed between serum selenium levels and body composition with r = correlation coefficient and with a significance level of *p* < 0.05.

**Table 6:** Spearman correlation between serum selenium level and thyroid hormone levels at different points in time within one year after breast cancer diagnosis.

Thyroid Hormones	Selenium Level at Baseline	Selenium Level after 3 Months	Selenium Level after 6 Months	Selenium Level after 9 Months	Selenium Level after 12 Months
TSH	r = −0.07 *p* = 0.51	r = −0.13 *p* = 0.26	r = −0.09 *p* = 0.43	r = 0.02 *p* = 0.88	r = 0.00 *p* = 0.98
T3	r = 0.11 *p* = 0.30	r = −0.14 *p* = 0.20	r = 0.05 *p* = 0.65	r = 0.11 *p* = 0.37	r = 0.01 *p* = 0.95
T4	r = 0.05 *p* = 0.67	r = 0.15 *p* = 0.17	r = 0.15 *p* = 0.20	r = −0.03 *p* = 0.82	r = −0.04 *p* = 0.77

Spearman correlation was performed between serum selenium levels and thyroid hormones with r = correlation coefficient and with a significance level of *p* < 0.05.

## Data Availability

The datasets generated during the current study are available from the corresponding author upon reasonable request.

## Abbreviations

BMI	body mass index
ER	estrogen receptor
PR	progesterone receptor
HER2	human epidermal growth factor 2
GEE	generalized estimating equations analysis
NST	no special type
UICC/AJCC	Union for International Cancer Control/American Joint Committee on Cancer
